# A novel phenotype in an Italian family with a rare progranulin mutation

**DOI:** 10.1007/s00415-022-11285-7

**Published:** 2022-07-20

**Authors:** Maria Claudia Russillo, Cristiano Sorrentino, Alfonso Scarpa, Claudia Vinciguerra, Giulio Cicarelli, Sofia Cuoco, Monica Gagliardi, Mariagrazia Talarico, Radha Procopio, Andrea Quattrone, Paolo Barone, Maria Teresa Pellecchia

**Affiliations:** 1 Center for Neurodegenerative Diseases (CEMAND), Department of Medicine, Surgery and Odontology “Scuola Medica Salernitana”, University of Salerno, Salerno, Italy; 2 Department of Medicine, Surgery and Dentistry, “Scuola Medica Salernitana”, University of Salerno, Salerno, Italy; 3 Neurology Unit, S.G. Moscati Hospital, Avellino, Italy; 4 Institute for Biomedical Research and Innovation, National Research Council, Cosenza, Italy; 5 Institute of Neurology, Department of Medical and Surgical Sciences, Magna Graecia University of Catanzaro, Catanzaro, Italy; 6 Department of Medical and Surgical Sciences, Neuroscience Research Center, Magna Graecia University of Catanzaro, Catanzaro, Italy

**Keywords:** Progranulin, *GRN* p.R298H mutation, Inspiratory stridor, Family history, Clinical variability

## Abstract

**Introduction:**

Progranulin (*PGRN*) is a secreted glycoprotein encoded in humans by the *GRN* gene, located on chromosome 17q21. Several nonsense and missense pathogenetic *GRN* mutations have been described.

**Objective:**

We herein describe two sisters carrying a rare *GRN* mutation with extremely different clinical features and family history of dementia and behavioral disorders, with a novel presentation with stridor and dysphonia.

**Methods:**

Patients underwent a multidimensional assessment including neurological and neuropsychological evaluation, structural and functional imaging, and genetic screening.

**Results:**

The younger sister presented at the age of 64 with inspiratory stridor, dysphonia and exercise-induced dyspnea. Transnasal fiberoptic laryngoscopy showed bilateral adduction of the vocal cords at rest and paradoxical further adduction of the vocal cords during forced inspiration, suggesting the hypothesis of an adductor laryngeal dystonia. The older sister presented at the age of 63 with a rapidly progressive corticobasal syndrome. The only clinical feature common to both sisters was a dysexecutive syndrome. The c.893G > A mutation in exon 9 of *GRN* was found in heterozygosis in both sisters, causing a missense Arginine to Histidine substitution in position 298 of the protein (p.R298H).

**Conclusions:**

Our report supports the pathogenicity of the *GRN* p.R298H mutation, which is first detected in two members from the same family, showing an extremely different phenotypes. Moreover, we report the first case of an FTD-associated mutation presenting with inspiratory stridor and dysphonia linked to adductor laryngeal dystonia, thus expanding the clinical spectrum of GRN-related disorders.

**Supplementary Information:**

The online version contains supplementary material available at 10.1007/s00415-022-11285-7.

## Introduction

Progranulin (*PGRN*) is a secreted glycoprotein encoded in humans by the *GRN* gene, located on chromosome 17q21. *PGRN* is a growth factor involved in numerous processes (e.g. central and peripheral nervous system development, wound healing, immune regulation and inflammation) and implicated also in tumorigenesis [1–3].

To date, several nonsense and missense pathogenetic *GRN* mutations have been described; known *GRN* mutations are collated in www.molgen.ua.ac.be/FTDmutations/. Heterozygous *GRN* mutations are linked to familial frontotemporal lobar degeneration (FTLD), whereas homozygous ones lead to the development of neuronal ceroid lipofuscinosis (NCL) [4]. Most of the currently known *GRN* pathogenic mutations introduce a premature stop codon that triggers nonsense-mediated loss of GRN mRNA and subsequent loss of 50% of plasma *PGRN* levels leading to haploinsufficiency. [5]

We herein describe neurological features of two sisters carrying a *GRN* mutation with extremely different clinical phenotypes and family history of dementia and behavioral disorders.

## Material and methods

### Patients

Patient 1 is a 65-year-old Caucasian woman followed at the Movement Disorder outclinic of the University of Salerno, Italy. Symptoms onset occurred at 64 years with dysphonia, exercise-induced dyspnea and inspiratory stridor, which was mainly nocturnal (Supplementary Audio file). Since stridor was her main complaint, she was referred to our Movement Disorders outclinic in order to consider a diagnosis of multiple system atrophy (MSA). Two paternal aunts were diagnosed with dementia and behavioral disorders. Her father died at 54 years due to complications of diabetes and did not present during lifetime any cognitive or behavioral symptoms. Her mother died in old age and did not present any neurological symptoms.

Patient 2 is a 68-year-old Caucasian woman that presented at the age of 63 with severe depression of mood, unresponsive to several antidepressants, and severe apathy.

### Investigations

Patient 1 performed DaT-SCAN, brain ^18^F-FDG PET, 3-Tesla brain MRI, chest CT, transnasal fiberoptic laryngoscopy, electromyography (EMG) and polysomnography. To better assess dysphonia, voice quality was evaluated by the acoustic voice quality index (AVQI), a tool also validated in Italian (Validation of the Acoustic Voice Quality Index (AVQI) version 03.01 in Italian [6], that quantifies the overall voice quality using concatenated 3 s of a sustained vowel [a:] and voice segments from a phonetically balanced text. The AVQI is a six-variable acoustic model that include smoothened cepstral peak prominence, shimmer local, shimmer local dB, slope of the spectrum, harmonics-to-noise ratio, tilt of the regression line through the spectrum according to the following formula: AVQI = [3.295 − (0.111*CPPs) − (0.073*HNR) − (0.213*shimmer local) + (2.789*shimmer local dB) − (0.032*slope) + (0.077*tilt)] *2.571 [7]. The voice sample was recorded in a quiet environment with low levels of ambient noise (signal-to-noise ratio > 30 dB) using a microphone Shure SM48 (Shure Incorporated Product Support Niles, IL) connected to a MacBook Pro computer (Apple, Cupertino, CA) and recording three seconds of a sustained vowel [a:] and five phonetically balanced sentences of the Italian version of the CAPE-V (55 syllables) at comfortable pitch and loudness [8]. The text file of the script AVQI version 02.03 (Maryn Y. De Acoustic Voice Quality Index in het software Praat: een praktische handleiding. Belsele: Vlaamse Vereniging voor Logopedisten; 2013) was run using Praat software. The recorded voice samples were loaded on the Praat software (Version 6.2.09 for Mac, Paul Boersma and David Weenink; Institute of Phonetic Sciences, University of Amsterdam, The Netherlands) using the settings mono channel, sampling frequency of 44.1 kHz, and 16-bit resolution.

Patient 2 underwent neuropsychological evaluation and brain MRI one year after the onset of behavioral symptoms.

### Genetic analysis

All 13 exons of the *GRN* gene, including the intron–exon boundaries, were PCR-amplified, and sequenced on an ABI 3500 Genetic Analyzed (Life Technologies, Carlsbad, CA, USA). To evaluate the evolutionary amino acidic conservation was performed the bioinformatics analysis. In silico analyses were performed to evaluate the pathogenic role of the c.893G > A using PholyPhen2 (http://genetics.bwh.harvard.edu/pph2/), Mutation Taster (http://www.mutationtaster.org/). MAPT, C9orf72, FUS, TARDBP, VCP and CHMP2B genes were also analyzed by Sanger Sequencing.

## Results

### Neurological and neuropsychological examination

At our first evaluation, one year after symptoms onset, patient 1 presented frontal release signs (i.e., palmar grasp reflex and sucking reflex), laryngeal stridor, moderate dysphonia, tetrahyperreflexia and left Babinski sign. No extrapyramidal signs, muscle weakness or atrophy were observed. The patient was clinically re-evaluated 6 months after the first assessment, and her neurological examination was unchanged. She underwent a comprehensive neuropsychological assessment, showing a dysexecutive syndrome with significantly impaired inhibitory control (Stroop test) and planning (copy of the Rey’s complex figure). Mini-Mental State Examination (MMSE) and Montreal Cognitive Assessment (MoCA) scores were in the normal range. Moreover, memory, visuo-spatial and language domains were preserved; no psychiatric symptoms were present. A single-domain non-amnesic mild cognitive decline was diagnosed.

As for patient 2, at onset of behavioral symptoms, her neurological examination was normal. One year later, she developed memory deficits and a clearly asymmetric parkinsonism with dysarthria, reduction of verbal fluency, spastic laughter and ideomotor apraxia. Levodopa therapy was tried, with poor response. Her neuropsychological examination revealed a severe dysexecutive syndrome and a multidomain cognitive decline. At the age of 65 years, she was anarthric and had developed severe dystonia on the left side of the body and in the cranial district. She was bedridden since the age of 66 years. She was diagnosed with corticobasal syndrome. At last examination at the age of 68, she presented severe dementia with few residual non-verbal emotional reactions, severe rigidity prevalent on the left side of the body, severe dysphagia requiring a semi-liquid diet.

### Investigations

**Fig. 1:**
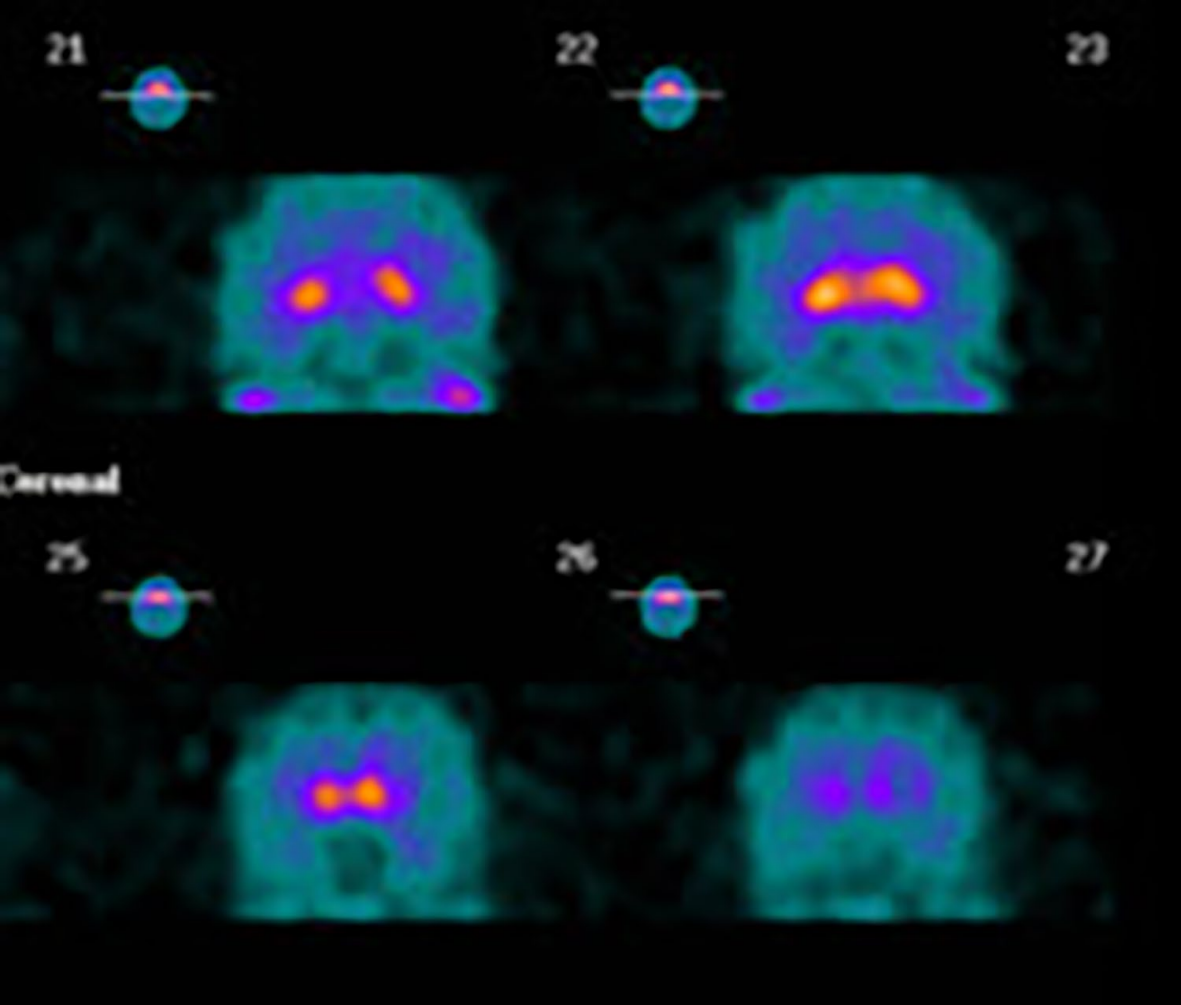
DaT-SCAN findings: reduced uptake in the right putamen

**Fig. 2:**
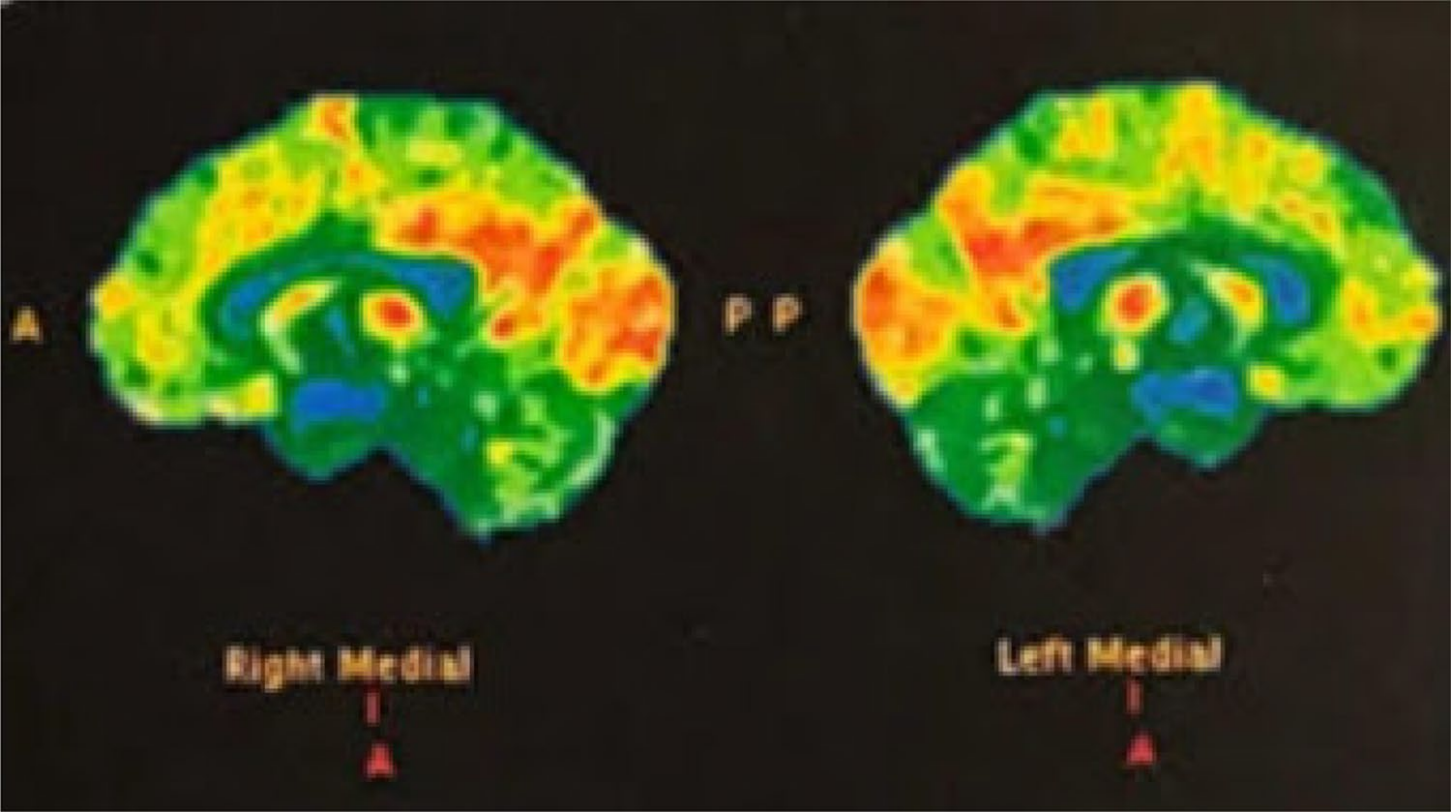
Brain ^18^F-FDG PET findings: hypometabolism in the medial frontal cortex

In Patient 1, DaT-SCAN showed reduced uptake in the right putamen (Fig. 1); brain ^18^F-FDG PET showed hypometabolism in the medial frontal cortex (Fig. 2); a 3-Tesla brain MRI was normal.

Chest CT allowed to exclude focal lesions obstructing or compressing the airways.

A transnasal fiberoptic laryngoscopy was performed to investigate the nature of the laryngeal stridor and exercise-induced dyspnea; at rest it showed bilateral adduction of the vocal cords in a paramedian position. No vocal fold structural lesion was found, and complete glottic closure during full phonation of *eee* (adductor task) was observed. Stroboscopy showed mild bilateral vocal tremor with a good lamina propria expansion without glottal gaps. During the forced inspiratory maneuver (abduction task), a paradoxical further adduction of the vocal cords was seen with airflow limitation due to glottic respiratory space reduction.

**Fig. 3:**
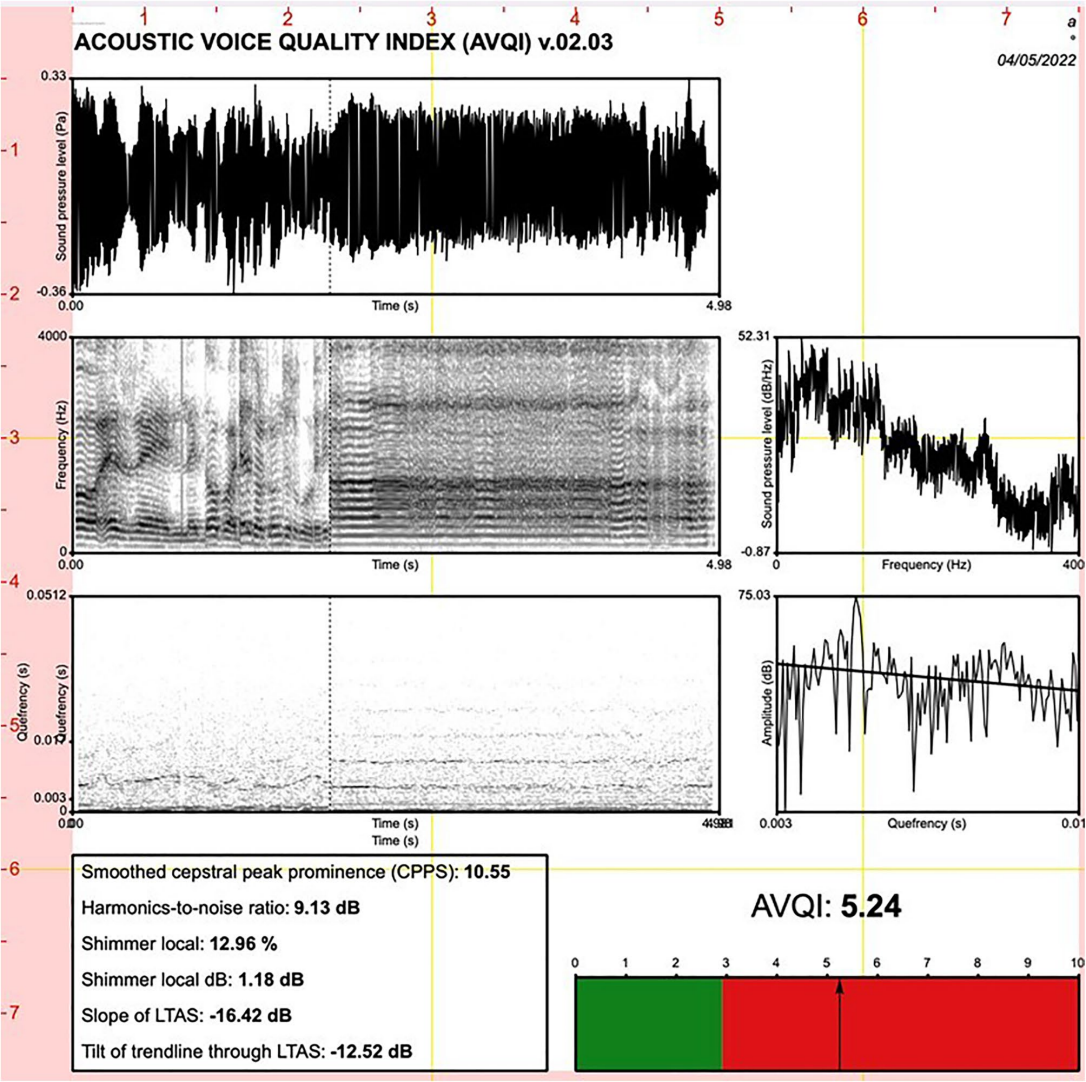
Graphical illustration of AVQI of patient III: **A** Oscillogram: representation of the concatenated vowel and sentence indicating the evolution of the sound pressure over time; **B** Spectrogram: visual representation of the spectrum of frequencies of a signal over time; **C** Long term average spectrum: logarithmic power spectral density which provides information on the spectral distribution of the speech signal over a period of time; **D** Power-Cepstrogram: shows cepstral splices as a function of time; **E** Power-Cepstrum: shows the tilt line of the power-cepstrum

An illustration of the patient's AVQI is shown in **Fig. ****3**. The optimal cut-off AVQI to discriminate between normophonic and dysphonic voices for Italian people was < 2.35, with a sensitivity of 90% and a specificity of 92% [9]. In our patient, the AVQI score was 5.24 and therefore it was considered abnormal. Polysomnography revealed obstructive apneas and hypopneas with an Apnea Hypopnea Index of 7 and a diagnosis of mild OSAS.

Needle EMG, performed in the bulbar, thoracic regions and at least two muscles innervated by different roots and peripheral nerves for each limb, showed widespread fasciculation potentials in the lumbosacral district and isolated fasciculation potentials in the cervicobrachial district, without chronic motor unit changes or reduced central activation. Central Motor Conduction, measured in the ulnar and posterior tibial nerves, resulted normal.

Brain MRI, performed in patient 2 one year after the onset of behavioral symptoms, was normal. Since she was rapidly bedridden, no further imaging studies were performed.

### Genetic analysis

**Fig. 4:**
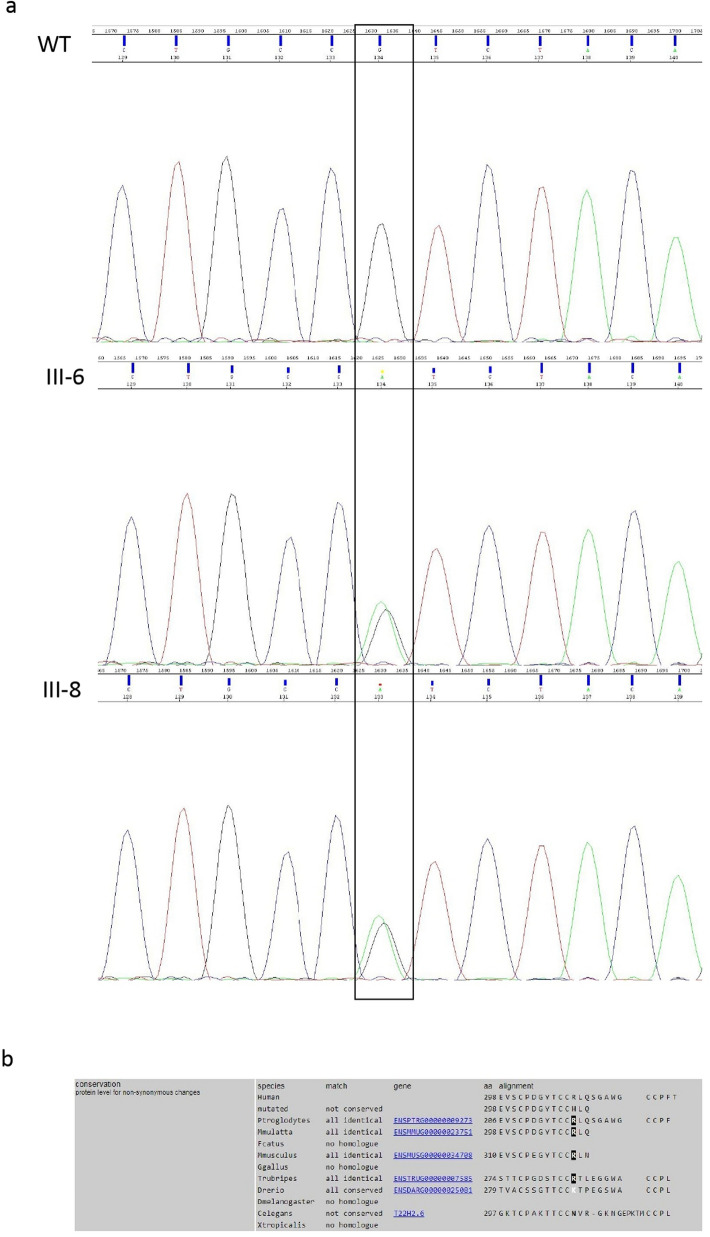
**a** Electropherogram shows the wildtype sequence (above) and the c.893G > A variant in heterozygous state (below); **b** alignment of GRN proteins shows high evolutionary conservation of Arginine 298

The c.893G > A variant in exon 9 of *GRN* was found in heterozygosis in both sisters (Fig. 4a). This variant, rs750810467, was very rare in genomic databases (frequency in GnomAD 0.00004775) and was not detected in 300 healthy subjects. The c.893G > A causes a missense Arginine to Histidine substitution in position 298 of the protein (p.R298H). The bioinformatic analysis showed that Arginine in position 298 is highly conserved across species (Fig. 4b). Functional prediction analysis by PolyPhen2 and Mutation Taster revealed that this variant has a probable damaging role. Furthermore, the mutational screening of *GNR* gene has highlighted the presence of one intronic variant (rs5848) in only one of the sisters. The presence of mutations in the MAPT, C9orf72, FUS, TARDBP, VCP and CHMP2B genes was also excluded in both sisters.

## Discussion

In this paper, we describe two sisters with quite different neurological phenotypes related to a rare GRN mutation. Sanger sequencing revealed in both sisters a missense variant (c.893G > A) not present in a control cohort of 300 healthy individuals of the same geographic area. Among rarely reported missense mutations, *GRN* p.R298H mutation was first described in 2010 in a single patient affected by FTLD with an unknown family history [10]. In 2016, the same mutation was reported in one FTLD patient with a substantial family history of dementia. [11]

The p.R298H is highly conserved across species. *Functional prediction analysis by PolyPhen2* (http://genetics.bwh.harvard.edu/pph2/) *and Mutation Taster* (http://www.mutationtaster.org/) *revealed that this variant has a probable damaging role.*

The p.R298H variant was already described by Yu et al. [10] as potentially pathogenic since not found in 760 healthy controls*.* The mutation was detected in only one patient with pathologically confirmed FLTD with ubiquitin-positive inclusions and unknown family history. [10]

The same variant was also reported by Karch et al*.* [11] in one FTLD patient presenting at 71 years with a typical corticobasal syndrome and a family history of dementia. A functional study showed that the p.R298H variant produced a moderate effect on PGRN secretion. [11]

Our data support the pathogenic role of *GRN* p.R298H variant in FTD, since it was first found in two Italian sisters with FTD and family history of behavioral disturbances and dementia.

Moreover, our findings suggest that this mutation may be associated with an extremely variable phenotype. In fact, the older sister showed a rapidly progressive corticobasal syndrome and was bedridden within three years from onset. A corticobasal syndrome with asymmetric akinetic rigid parkinsonism, asymmetric upper limb dystonia and cortical signs (echolalia, grasping, denomination and memory deficits, left hand apraxia) similar to that observed in patient 2 was reported by Karch et al. in association with the same mutation. [11]

In the younger sister, the main complaints were stridor and exercise-induced dyspnea. To the best of our knowledge, stridor has never been described in patients with frontotemporal dementia or *GRN* mutations. Indeed, inspiratory stridor is a typical feature of multiple system atrophy, where it is considered a diagnostic red flag, but our patient did not present parkinsonian or cerebellar signs, despite a moderately reduced DaT uptake in the right putamen. In MSA, the pathogenesis of stridor is debated, and the two mechanisms most likely involved are the degeneration of the ambiguous nucleus, found in some pathological studies, and the paradoxical muscular activity of the laryngeal adductor muscles during inspiration, found by EMG examination during sleep [12]. In our patient, laryngoscopy support the hypothesis of an adductor laryngeal dystonia (ADLD), manifesting with inspiratory stridor, dysphonia and exercise-induced dyspnea [13]. Indeed, ADLD is characterized by persistent inspiratory stridor and recurrent breathing difficulties, with paradoxical vocal cord movements on inspiration. [14]

A study of patients with a clinical diagnosis of ADLD and Abductor laryngeal dystonia was performed to assess the neural correlates of abnormal sensory discrimination by means of fMRI. In both groups, abnormal temporal discrimination thresholds were related with brain activation during symptom production in the left primary sensorimotor cortex and with resting brain activation in the left anterior cingulate cortex. ADLD patients showed negative correlations between abnormal temporal discrimination thresholds and symptom-related brain activation in the left middle/inferior gyrus, posterior cingulate cortex and bilateral SMA, while the left superior frontal gyrus and precuneus were positively correlated during the resting state [15]. We can speculate that frontal cortex impairment, documented in our patient by both neuropsychological assessment, showing a dysexecutive syndrome, and FDG-PET showing medial frontal hypometabolism, may be involved in the development of adductor laryngeal dystonia and stridor. Moreover, neuropsychological findings are consistent with the FDG-PET hypometabolism in the medial frontal cortex, since the performance of the Stroop test in healthy subjects corresponds to an fMRI activation in the anterior cingulate cortex. [16] As for the altered performance on the copy task of the Rey’s complex figure, that is a planning task, dorsolateral frontal cortex and its functional and structural connections with the anterior cingulate cortex may be involved. [17]

Moreover, the EMG findings of patient 1 met the El Escorial criteria for a “clinically possible ALS”, suggesting a subtle MN degeneration associated with FTLD, confirming the wide phenotypic variability of FTLD mutations [18], and requiring of a longer follow-up. Indeed, as reported in previous studies, [19, 20] a sizable percentage of FTLD patients show signs and symptoms of MN dysfunction, without progression to defined ALS over the time.

## Conclusions

Stridor has shown a high positive predictive value for a diagnosis of MSA, but it cannot be considered pathognomonic for this disease. Our patient was referred to us with a suspicion of MSA, but both clinical features and positive family history, albeit with different clinical manifestations, prompted us to search for an alternative diagnosis. Our paper expands the spectrum of clinical symptoms of FLTD with the first ever described case of stridor associated with a FLTD mutation. This rare GRN mutation seems to be associated with a variable phenotype at onset, but also with a different disease progression. Additional genetic studies in larger Italian cohort are required to clarify the role of this variant in the development of disease.

## Supplementary Information

Below is the link to the electronic supplementary material.

Supplementary file1 (MP4 28387 KB)

Supplementary file2 (MP4 526 KB)
